# Supplementary material for: Synthesis, Biological Activity, and Molecular Dynamics Study of Novel Series of a Trimethoprim Analogs as Multi-Targeted Compounds: Dihydrofolate Reductase (DHFR) Inhibitors and DNA-Binding Agents
Source: Int J Mol Sci. 2021 Apr 1;22(7):3685. doi: 10.3390/ijms22073685 (PMC8037161; doi:10.3390/ijms22073685)

Supplementary materials Table S1. Analytical and spectral data of the synthesized compound.

No.	A	E	^{1}H NMR	^{13}C NMR	Yield [%]	Exact Mass Formula
**1**	**A_1** 2-iodo-4-nitroaniline (0,43g)	**E_1** 3,4,5-trimethoxybenzoyl chloride (0.37 g)	3.85 (s, 3H, OCH_3), 3.99 (s, 6H, $2OCH_3$), 6.88 (d, 1H, Ar-H), 7.20 (d, 1H, Ar-H), 7.45 (s, 2H, Ar-H), 8.05 (s,1H, Ar-H)	54.32 ($2OCH_3$), 59.11 (OCH_3), 111.8 (CH), 114.63 (CH), 117.46 (2CH), 120.28 (C), 122.94 (C), 125.08 (CH), 158.00 (C) 158.75 (2C), 159.16 (2C), 164.88 (CONH)	48	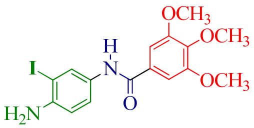 **$C_{16}H_{17}N_2O_4I$** [428.208]
**2**	**A_2** 2-fluoro-5-nitroaniline (0,26 g)	**E_1** 3,4,5-trimethoxybenzoyl chloride (0.37 g)	3.88 (s, 3H, OCH_3), 3.99 (s, 6H, OCH_3), 6.82 (d, 1H, Ar-H), 7.13 (s, 1H, Ar-H), 7.30 (s, 2H, Ar-H), 7.45 (d,1H, Ar-H)	54.35 ($2OCH_3$), 59.20 (OCH_3), 110.66 (2CH), 117.66 (2CH), 125.09 (CH), 128.01 (C), 130.64 (C), 133.84 (C), 145.49 (C), 159.38 (C), 159.75 (2C), 165.07 (CONH)	47	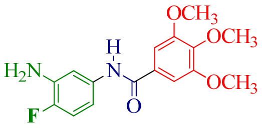 **$C_{16}H_{17}N_2O_4F$** [321.4]
**3**	**A_3** 4-fluoro-3-nitroaniline (0,26 g)	**E_1** 3,4,5-trimethoxybenzoyl chloride (0.37 g)	3.88 (s, 3H, OCH_3), 3.95 (s, 6H, $2OCH_3$), 6.72 (s, 1H, Ar-H), 6.88 (d, 1H, Ar-H), 7.22 (d, 1H, Ar-H), 7.45 (s, 2H, Ar-H)	54.36 ($2OCH_3$), 59.13 (OCH_3), 110.86 (2CH), 116.45 (2CH), 117.50 (CH), 123.04 (C), 124.10 (C), 125.09 (C), 145.49 (2C), 159.30 (2C), 165.01 (CONH)	47	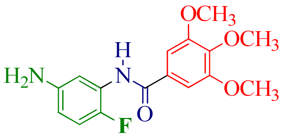 **$C_{16}H_{18}N_2O_4F$** [320.302]
**4**	**A_4** 2-chloro-4-nitroaniline (0,28g)	**E_1** 3,4,5-trimethoxybenzoyl chloride (0.37 g)	3.88 (s, 3H, OCH_3), 3.99 (s, 6H, $2OCH_3$), 6.88 (d, 1H, Ar-H), 7.18 (d, 1H, Ar-H), 7.45 (s, 2H, Ar-H), 7.95 (s, 1H, Ar-H),	54.35 ($2OCH_3$), 59,17 (OCH_3), 110.65 (CH), 116.43 (2CH),, 120.28 (C), 122.94 (C), 124.51 (C), 125.08 (CH), 130.64 (C), 145.47 (CH), 155.12 (C), 159.37 (2C), 164.97 (CONH)	51	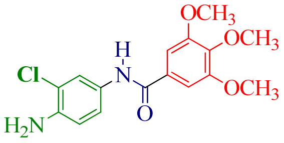 **$C_{16}H_{17}N_2O_4Cl$** [336.756]

**5**	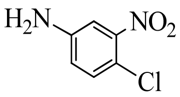 **A_5** 4-chloro-3-nitroaniline (0,28g)	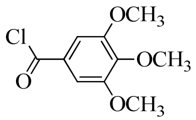 **E_1** 3,4,5-trimethoxybenzoyl chloride (0.37 g)	3. 68 (s, 3H, OCH_3), 3.95 (s, 6H, $2OCH_3$), 7.45 (s, 2H, Ar-H), 7.65 (d, 1H, Ar-H), 7.78 (d, 1H, Ar-H), 7.98 (s, 1H, Ar-H),	54.36 ($2OCH_3$), 59.27 (OCH_3), 110.86 (2CH), 116.45 (2CH), 117.50 (CH), 123.04 (C), 124.10 (C), 125.09 (C), 145.49 (2C), 159.30 (2C), 165.04 (CONH)	52	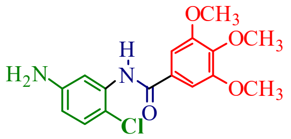 **$C_{16}H_{17}N_2O_4Cl$** [336.756]
**6**	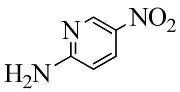 **A_6** 2-amino- -5-nitropyridine (0,23g)	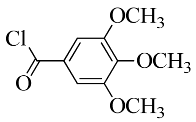 **E_1** 3,4,5-trimethoxybenzoyl chloride (0.37 g)	3.86 (s, 3H, OCH_3), 3. 95 (s, 6H, $2OCH_3$), 6.80 (d, 1H, Py-H), 7. 27 (s, 2H, Ar-H), 7.44 (d, 1H, Py-H), 7.98 (s, 1H, Py-H)	56.77 ($2OCH_3$), 61.15 (OCH_3), 106.27 (2CH), 116.32 (CH), 124.61 (CH), 131.38 (C), 131.58 (C), 142.19 (C), 154.47 (2C), 156.09 (C), 168.06 (CONH)	45	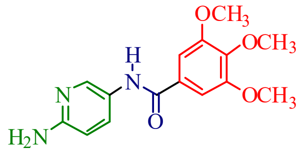 **$C_{15}H_{17}N_3O_4$** [303.300]
**7**	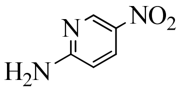 **A_6** 2-amino- -5-nitropyridine (0,23g)	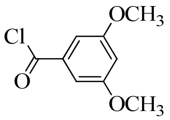 **E_2** 3,5-dimethoxybenzoyl chloride (0.32 g)	3.87 (s, 6H, OCH_3), 6.67 (s, 1H, Ar-H), 6.79 (dd, 1H, Py-H), 7.17 (s, Py-H), 7.45 (s, 2H, Ar-H)	56.02 ($2OCH_3$), 104.61 (CH) 106.44 (2CH), 116. 42 (CH), 124.49 (CH), 131.44 (C), 138.32 (C), 155.88 (C), 162.43 (2C), 168.39 (CONH)	44	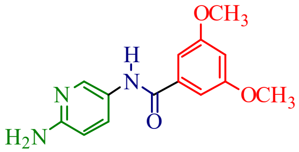 **$C_{14}H_{15}N_3O_3$** [273.275]
**8**	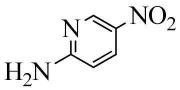 **A_6** 2-amino- -5-nitropyridine (0,23g)	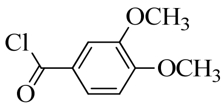 **E_3** 3,4-dimethoxybenzoyl chloride (0.32 g)	3.95 (s, 6H, OCH_3), 6.78 (dd, 2H, Py-H), 7.05 (d, 1H, Ar-H), 7. 43 (s, 1H, Py-H), 7. 52 (s, 1H, Ar-H), 7.57 (d, 1H, Ar-H)	56.48 (OCH_3), 61.53 (OCH_3), 112.03 (CH), 112.14 (CH), 116. 22 (CH), 116. 30 (CH), 122.15 (CH), 124.49 (CH), 131.64 (C), 150.29 (C), 153.60 (C), 155.72 (2C), 168.24 (CONH)	46	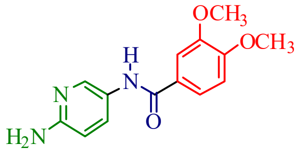 **$C_{14}H_{15}N_3O_3$** [273.275]
**9**	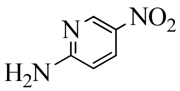 **A_6** 2-amino- -5-nitropyridine (0,23g)	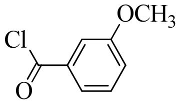 **E_4** 3- methoxybenzoyl chloride (0.27 g)	3.85 (s, 3H, OCH_3), 6.78 (dd, 2H, Py-H), 7.12 (d, 1H, Ar-H), 7.40-7. 52 (m, 3H, Ar-H), 7.99 (s, 1H, Py-H)	55.90 (OCH_3), 113.80 (CH), 112.03 (CH), 116.23 (CH), 118.55 (CH), 120.62 (CH), 122.99 (CH), 124.46 (CH), 131.52 (C), 131.89 (CH), 149.22 (C), 155.81 (C), 161.31 (C), 168.51 (CONH)	50	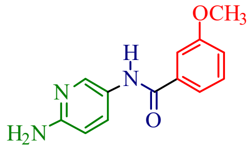 **$C_{13}H_{13}N_3O_2$** [243.160]

**10**	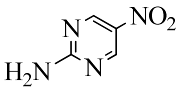 **A_7** 2-amino- -5-nitropyrimidine (0,23g)	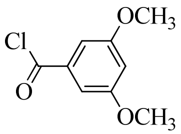 **E_1** 3,5-dimethoxybenzoyl chloride (0.32 g)	3.85 (s, 6H, OCH_3), 6.57 (s, 2H, Ar-H), 6.62 (s, 1H, Ar-H), 7.62 (s, 2H, Pyr-H)	55.64 (2OCH₃), 105.70 (CH), 106.39 (2CH), 121.06 (C), 122.67 (C), , 135.23 (C), 148.55 (2CH), 154.87 (2C), 168.51 (CONH)	55	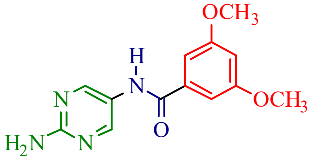 **$C_{13}H_{14}N_4O_3$** [274.263]
**11**	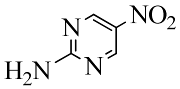 **A_7** 2-amino- -5-nitropyrimidine (0,23g)	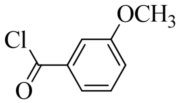 **E_1** 3- methoxybenzoyl chloride (0.27 g)	3.85 (s, 3H, OCH_3), 6. 92 (d, 1H, Ar-H), 6.94 (d, 1H,Ar-H), 7.54 (tr, 1H, Ar-H), 7.58 (d, 1H,Ar-H), 7.62 (s, 2H, Pyr-H)	56.86 (OCH_3), 114.94 (CH), 116.29 (CH), 117.61 (CH), 130.64 (CH), 131.63 (C), 137.23 (C), 149.41 (2CH), 155.68 (C), 161.26 (C), 169.39 (CONH)	56	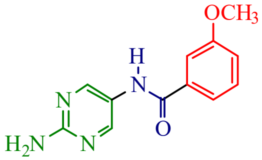 **$C_{12}H_{12}N_4O_2$** [244.239]
**12**	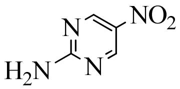 **A_7** 2-amino- -5-nitropyrimidine (0,23g)	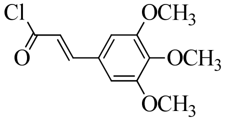 **E_5** 3,4,5-trimethoxy-trans-cinnamoyl chloride (0.45 g)	3.80 (s, 3H, OCH_3), 3.92 (s, 6H, OCH_3), 6.68 (d, 1H, CH), 6.94 (s, 2H, Ar-H), 7.55 (d, 1H, CH), 7.95 (s, 2H, Pyr-H)	56.68 (2OCH₃), 61.20 (OCH_3), 106.36 (2CH), 116.32 (2CH), 118.55 (CH), 121.84 (C), 123.10 (C),138.60 (C), 142.20 (CH), 154.85 (2C), 155.81 (C), 169.21 (CONH)	58	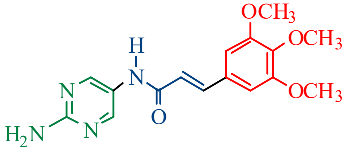 **$C_{16}H_{18}N_4O_4$** [330.235]
**13**	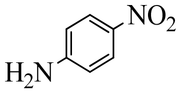 **A_8** 4-nitroaniline (0.23 g)	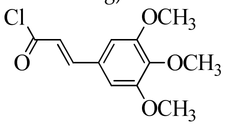 **E_5** 3,4,5-trimethoxy-trans-cinnamoyl chloride (0.45 g)	3.80 (s, 3H, OCH_3), 3.90 (s, 6H, OCH_3), 6.67 (d, 1H, CH), 6.78 (d, 2H, Ar-H), 6.94 (s, 2H, Ar-H), 7.45 (d, 2H, Ar-H), 7.55 (d, 1H, CH)	56.68 (2OCH₃), 61.19 (OCH_3), 106.35 (2CH), 116.34 (2CH), 116.37 (C), 118.55 (CH), 121.85 (C), 123.09 (CH), 132.26 (C), 142.19 (CH), 147.61 (C), 154.85 (2C), 166.39 (CONH)	60	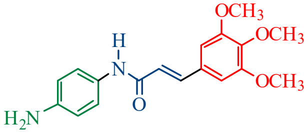 **$C_{18}H_{20}N_2O_4$** [304.327]
**14**	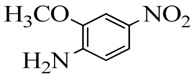 **A_9** 2-methoxy- -4-nitroaniline (0,28	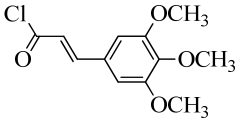 **E_5** 3,4,5-trimethoxy-trans-cinnamoyl chloride (0.45 g)	3.78 (s, 3H, OCH_3), 3.80 (s, 3H, OCH_3), 3.90 (s, 6H, OCH_3), 6.68 (d, 1H, CH), 6.78 (d, 1H, Ar-H), 6.92 (s, 2H, Ar-H), 7.44 (d, 2H, Ar-H), 7.56 (d, 1H, CH)	56.69 (2OCH₃), 59.10 (OCH_3), 61.20 (OCH_3), 106.38 (2CH), 116.35 (2CH), 118.55 (CH), 121.87 (C), 123.10 (CH), 132.26 (C), 140.89 (C), 142.19 (CH), 147.31 (C), 154.86 (2C), 166.40 (CONH)	44	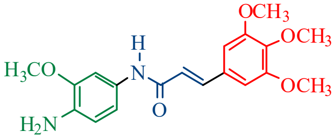 **$C_{19}H_{21}N_2O_5$** [357.4]

	g)					
**15**	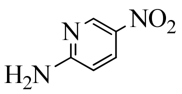 **A6** 2-amino- -5-nitropyridine (0,23g)	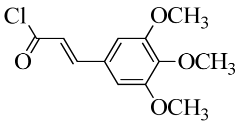 **E5** 3,4,5-trimethoxy-trans-cinnamoyl chloride (0.45 g)	3.88 (s, 3H, OCH_3), 3.91 (s, 6H, OCH_3), 6.75 (d, 1H, Py-H), 6.89 (s, 1H, CH), 7.27 (s, 2H, Ar-H), 7.45 (d, 1H, Py-H), 7.83 (d, 1H, CH), 7.98 (s, 1H, Py-H)	56.77 ($2OCH_3$), 61.15 (OCH_3), 106.27 (2CH), 116.32 (CH), 118.82 (CH), 124.61 (CH), 131.38 (C), 131.58 (C), 141.72 (CH), 142.19 (C), 154.07 (2C), 156.29 (C), 167.96 (CONH)	38	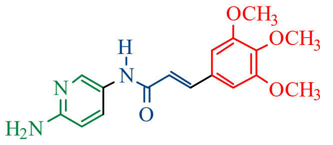 **$C_{17}H_{19}N_3O_4$** [329.4]
**16**	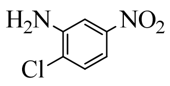 **A4** 2-chloro-5-nitroaniline (0,28g)	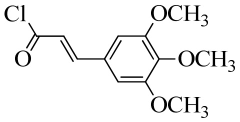 **E5** 3,4,5-trimethoxy-trans-cinnamoyl chloride (0.45 g)	3.85 (s, 3H, OCH_3), 3.88 (s, 6H, OCH_3), 6.65 (s, 1H, CH), 6.78 (s, 2H, Ar-H), 6.89 (d, 1H, Ar-H), 7.35 (d, 1H, Ar-H), 7.45 (d, 1H, Ar-H), 7.83 (d, 1H, CH)	55.64 ($2OCH_3$), 57.48 (OCH_3), 105.07 (CH), 115.07 (2CH), 117.64 (CH), 118.09 (CH), 121.28 (C), 122.67 (C), 129.68 (CH), 130.64 (C), 137.47 (2C), 141.20 (CH), 154.12 (C), 157.30 (C), 164.80 (CONH)	40	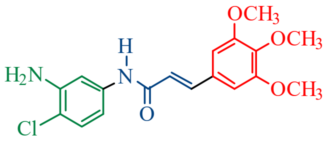 **$C_{19}H_{19}N_2O_4Cl$** [374.9]
**17**	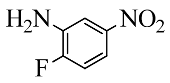 **A2** 2-fluoro-5-nitroaniline (0,26 g)	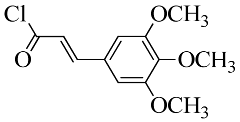 **E5** 3,4,5-trimethoxy-trans-cinnamoyl chloride (0.45 g)	3.82 (s, 3H, OCH_3), 3.88 (s, 6H, OCH_3), 6.45 (d, 1H, Ar-H), 6.55 (s, 1H, CH), 6.68 (s, 2H, Ar-H), 6.82 (d, 1H, Ar-H), 7.31 (d, 1H, Ar-H), 7.65 (d, 1H, CH)	56.77 ($2OCH_3$), 61.15 (OCH_3), 105.89 (2CH), 107.79 (CH), 116.33 (CH), 117.35 (CH), 118.02 (CH), 122.67 (C), 129.68 (C), 130.64 (C), 137.47 (C), 139.31 (CH), 143.12 (C), 154.67 (2C), 165.50 (CONH)	45	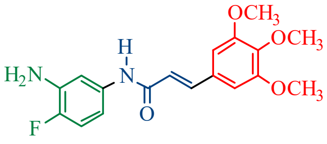 **$C_{19}H_{19}N_2O_4F$** [358.4]
**18**	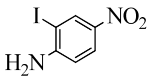 **A1** 2-iodo-4-nitroaniline (0,43g)	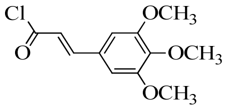 **E5** 3,4,5-trimethoxy-trans-cinnamoyl chloride (0.45 g)	3.82 (s, 3H, OCH_3), 3.89 (s, 6H, $2OCH_3$), 6.65 (d, 1H, CH), 6.88 (d, 1H, Ar-H), 7.25 (d, 2H, Ar-H), 7.55 (d, 1H, CH), 7.95 (s, 2H, Ar-H), 8.05 (s, 1H, Ar-H),	56.56 ($2OCH_3$), 61.15 (OCH_3), 105.87 (C), 106.89 (2CH), 107.29 (CH), 126.21 (CH), 128.78 (C), 129.52 (CH), 133.41 (C), 134.05 (CH), 139.93(C), 140.68 (CH), 153.73 (C), 154.65 (2C), 163.88 (CONH)	44	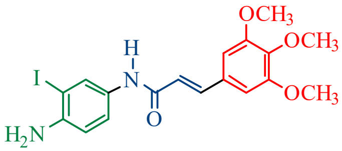 **$C_{19}H_{19}N_2O_4I$** [466.3]

Supplementary materials 2. 1H and 13 C NMR spectra of the new compounds **1–18.**

compound **1**

1H

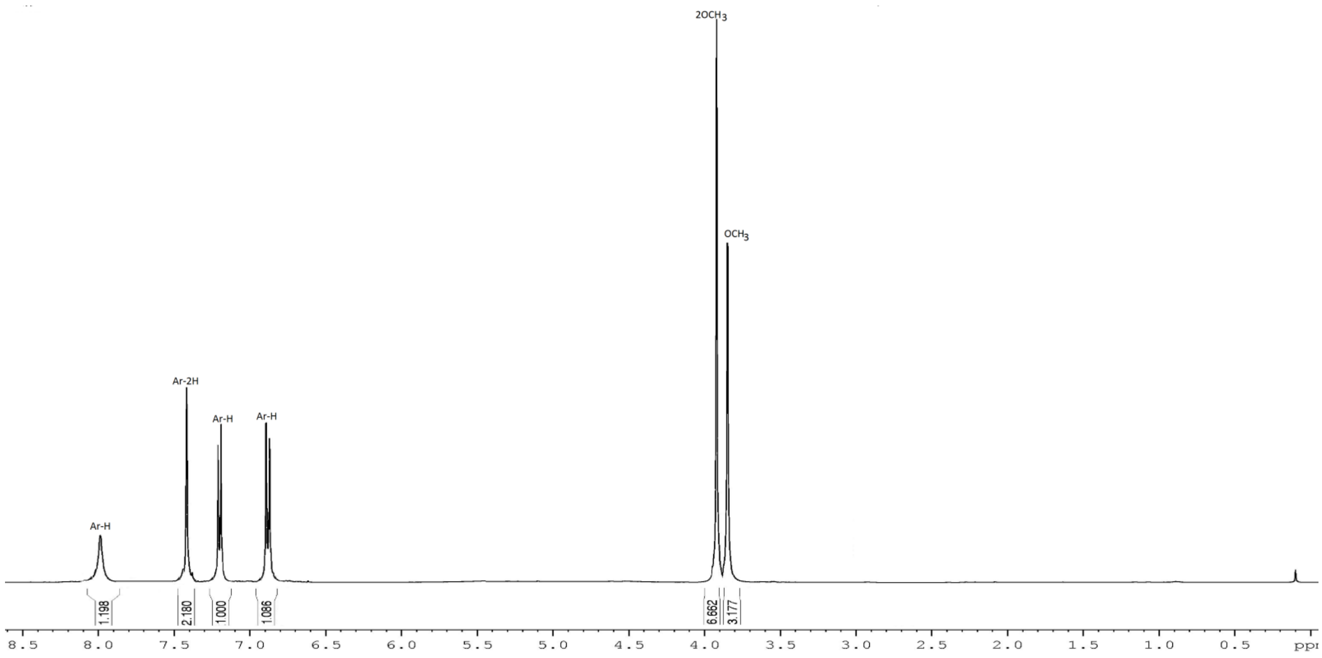

13C

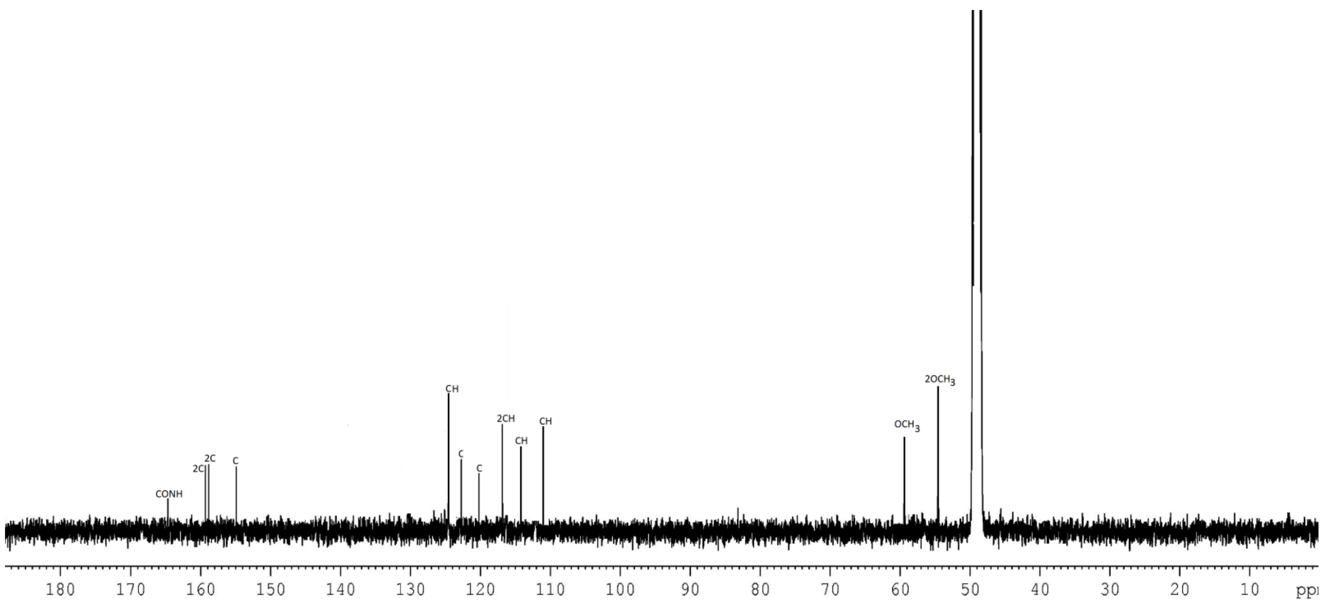

compound **2**

1H

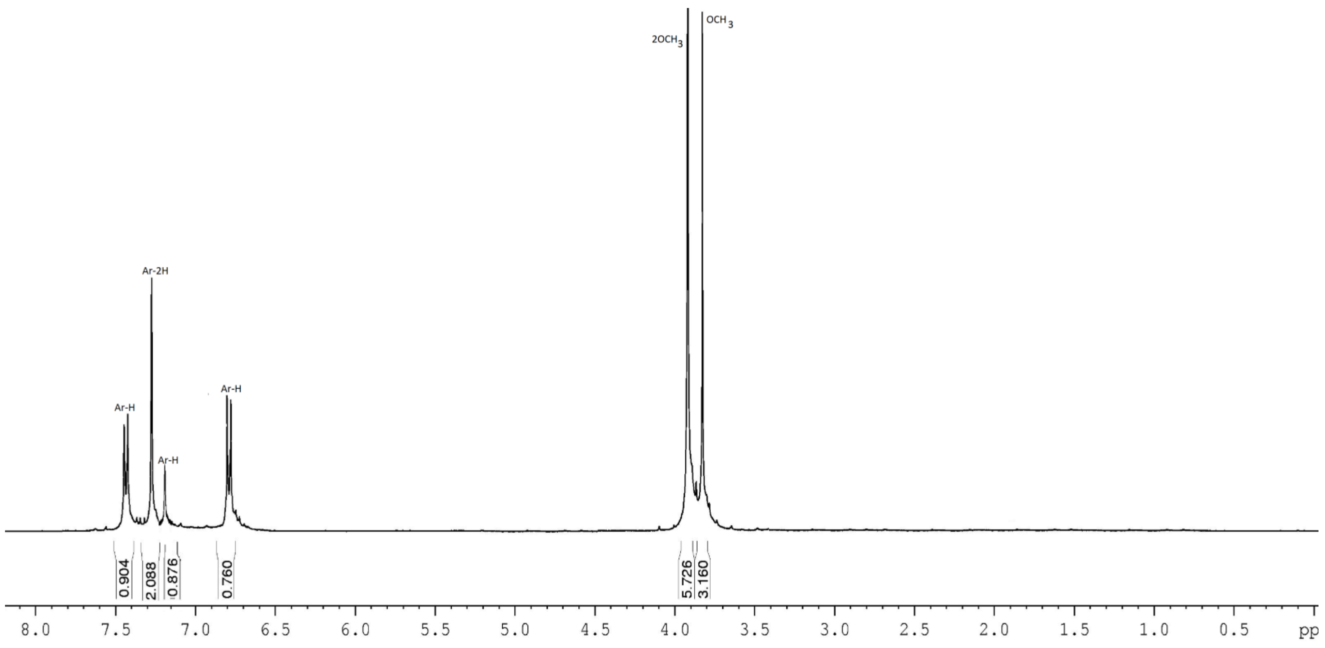

13C

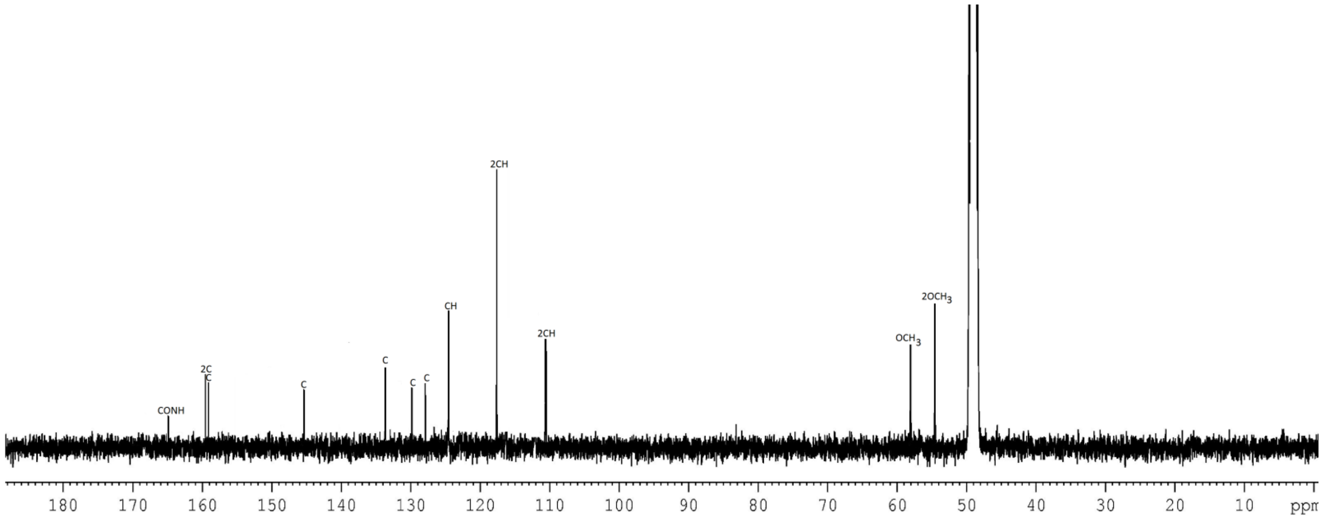

compound **3**

1H

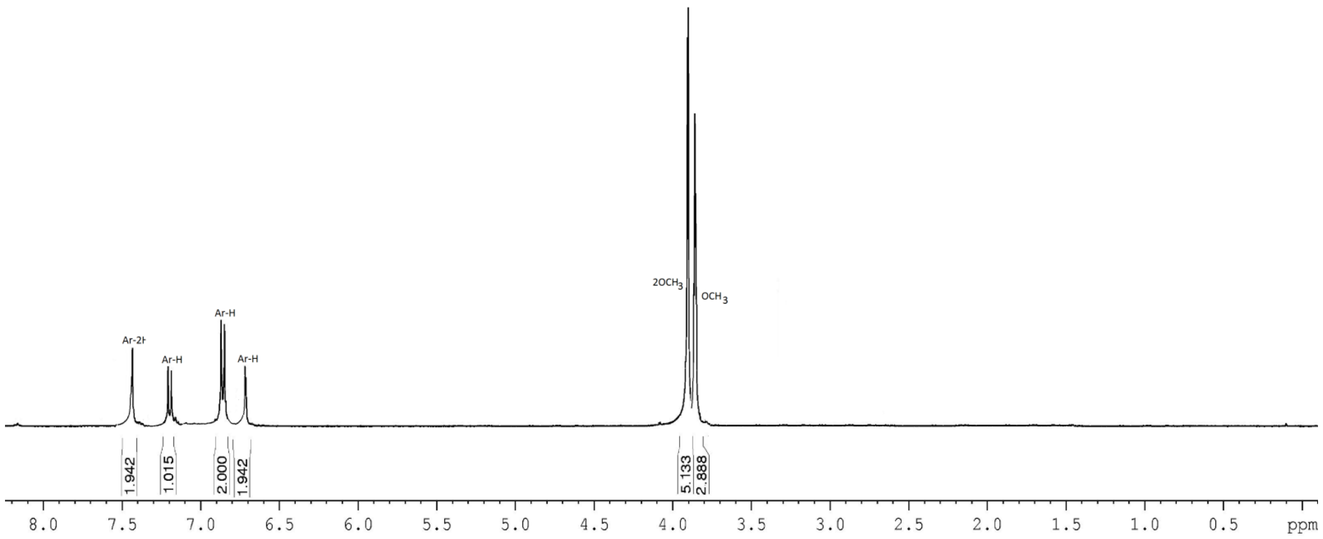

13 C

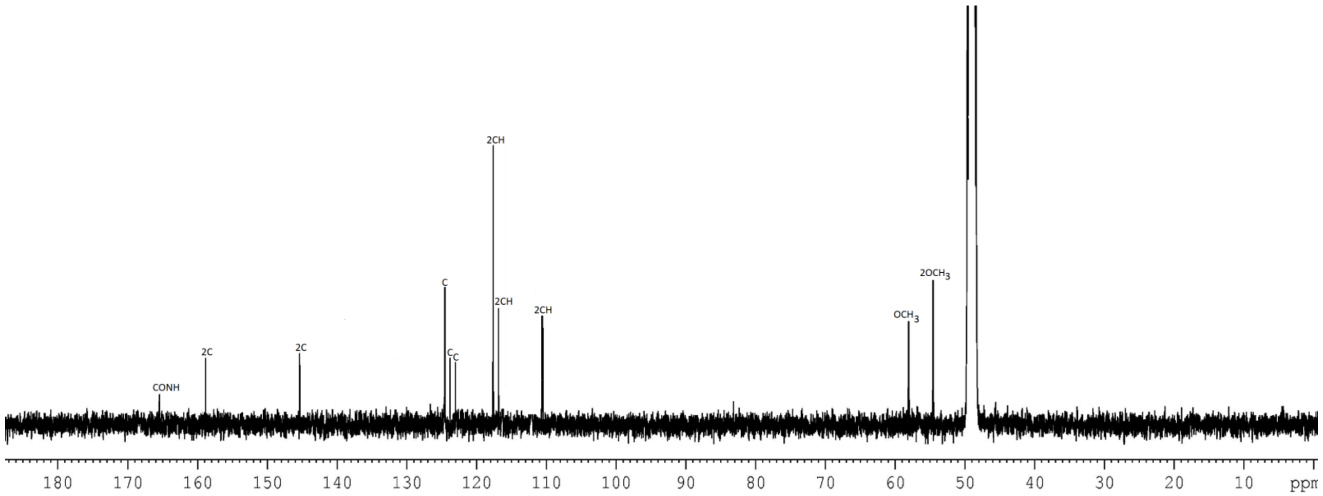

compound **4**

1H

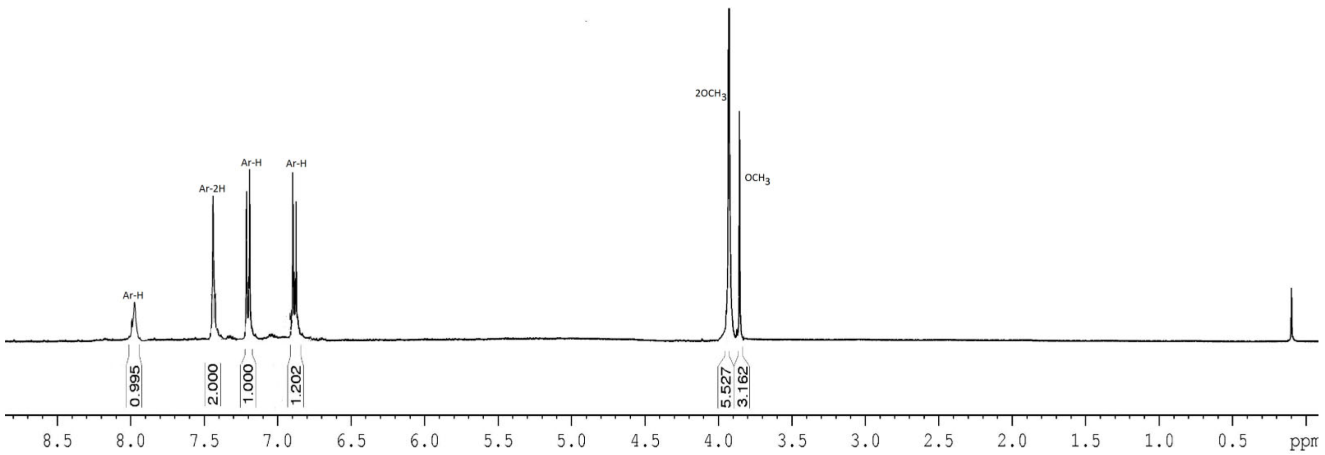

13C

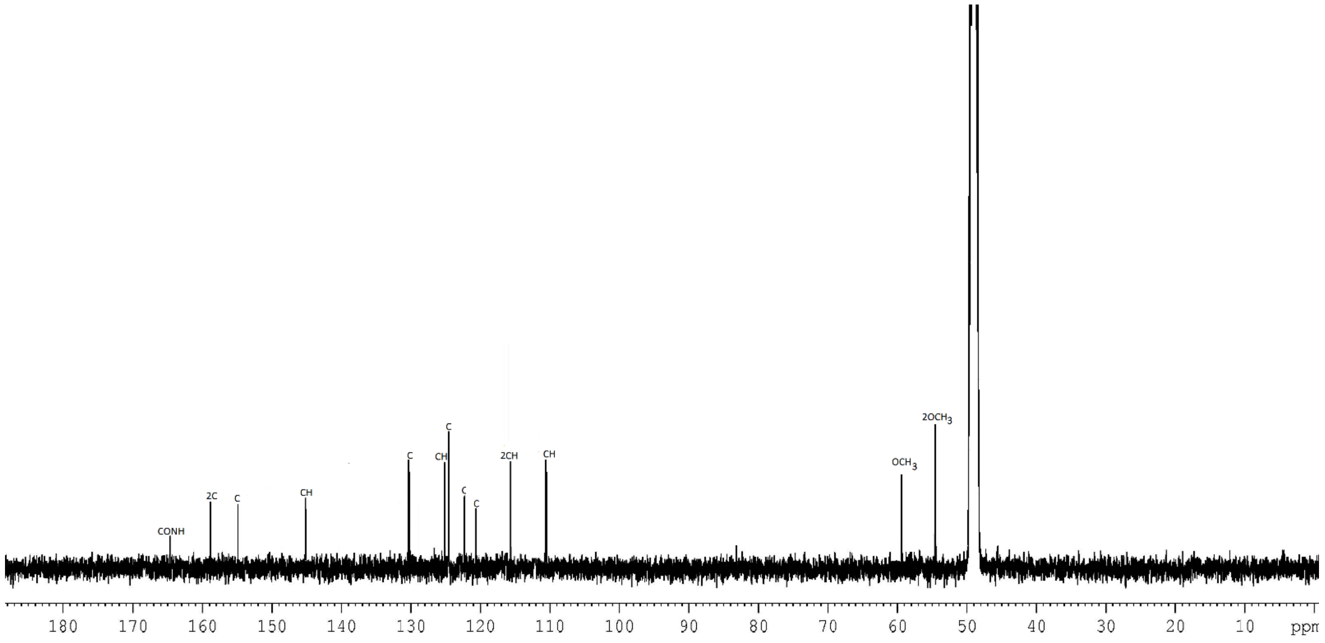

compound **5**

1H

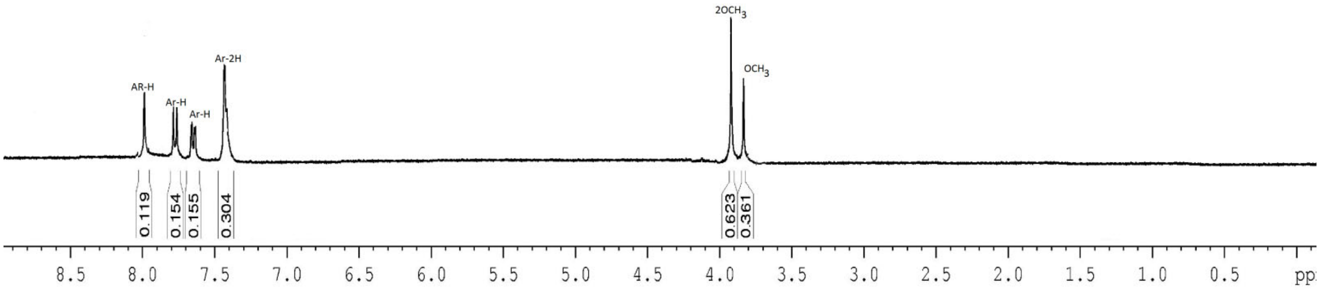

13C

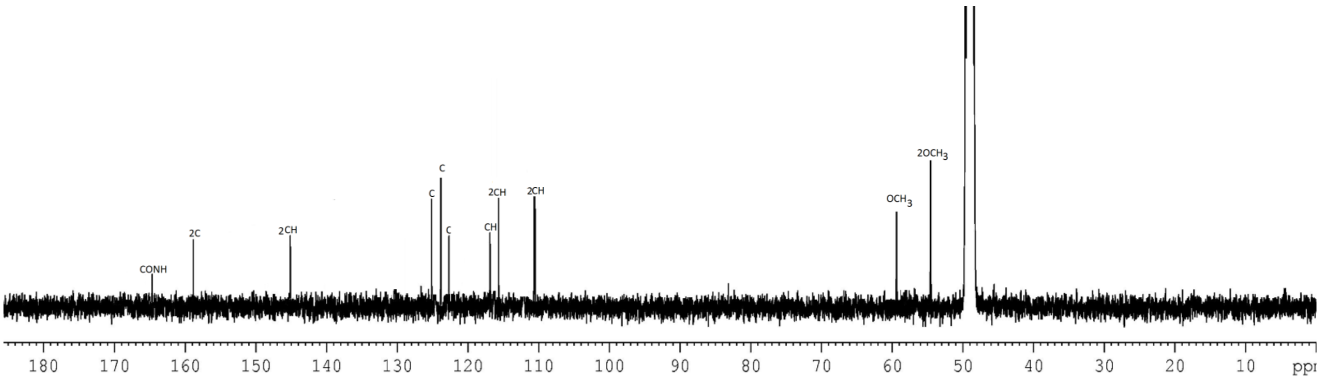

compound **6**

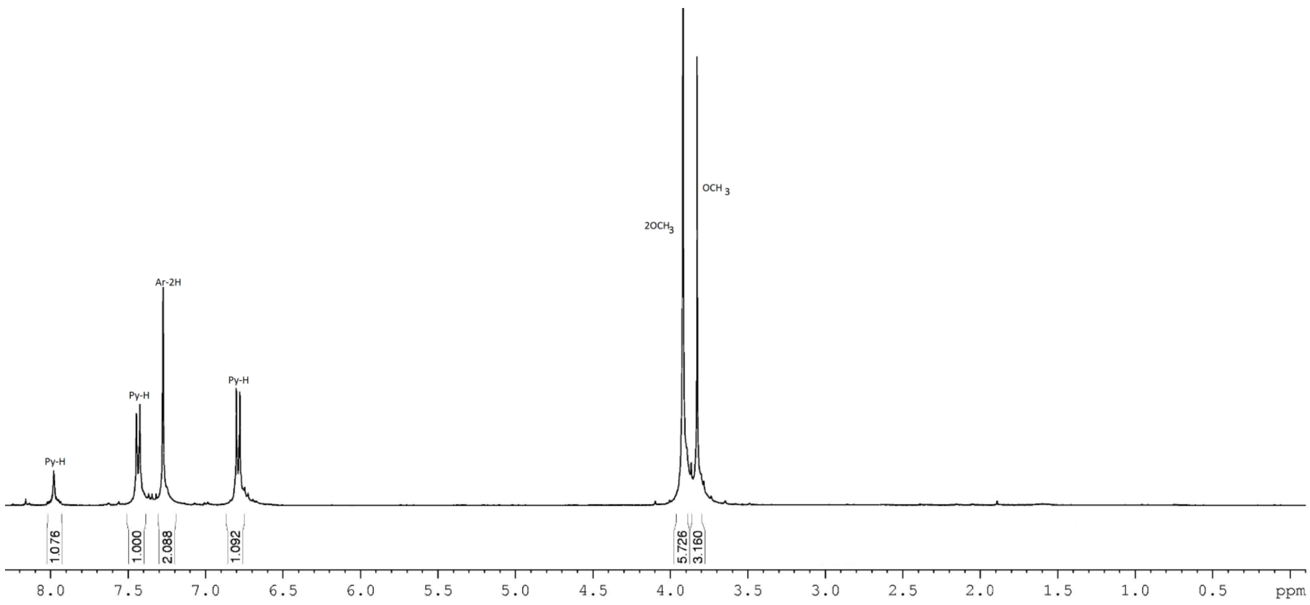

13 C

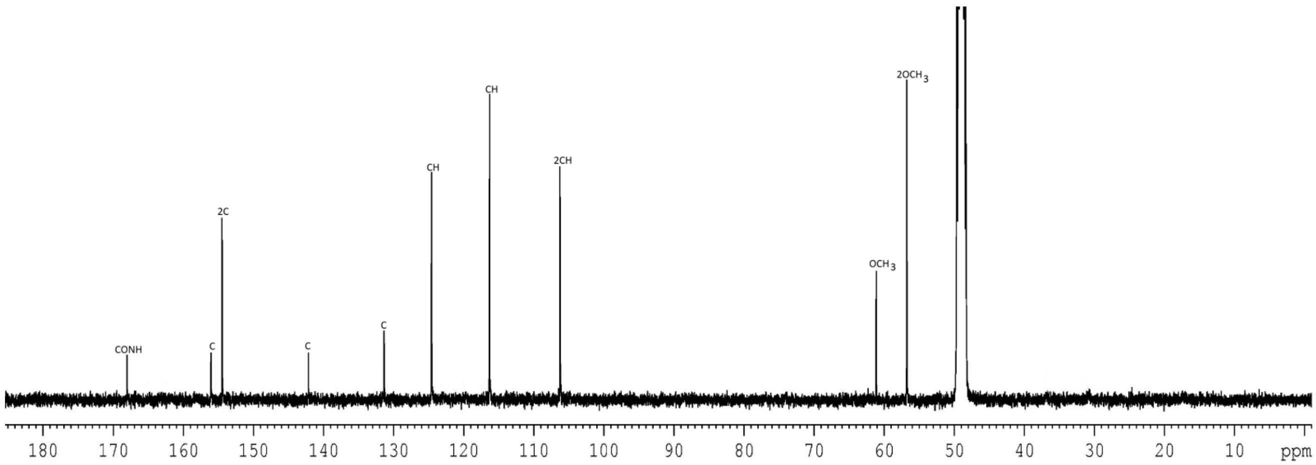

compound **7**

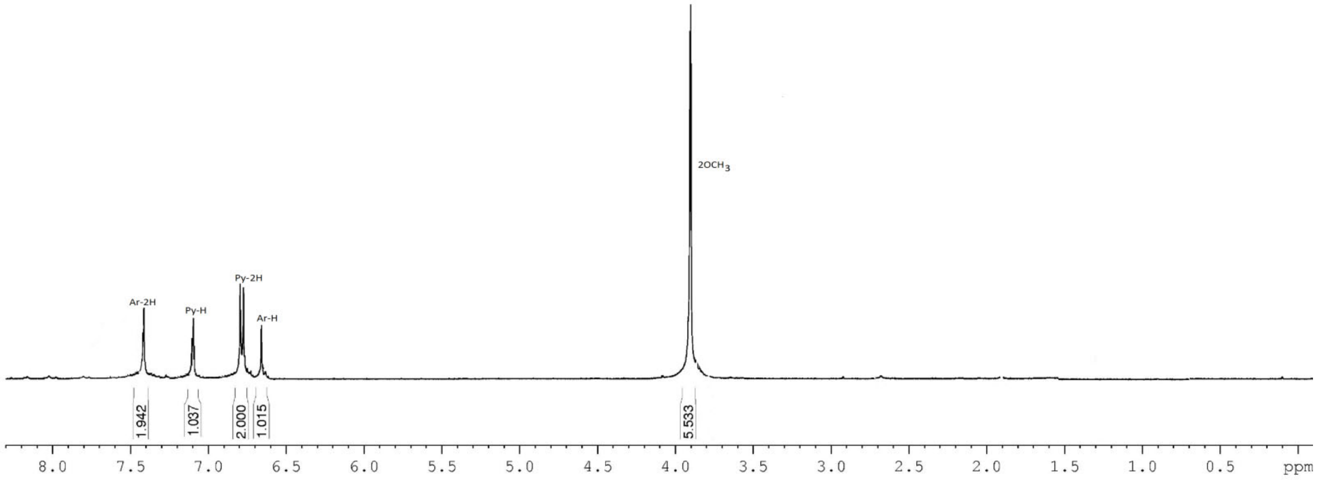

13C

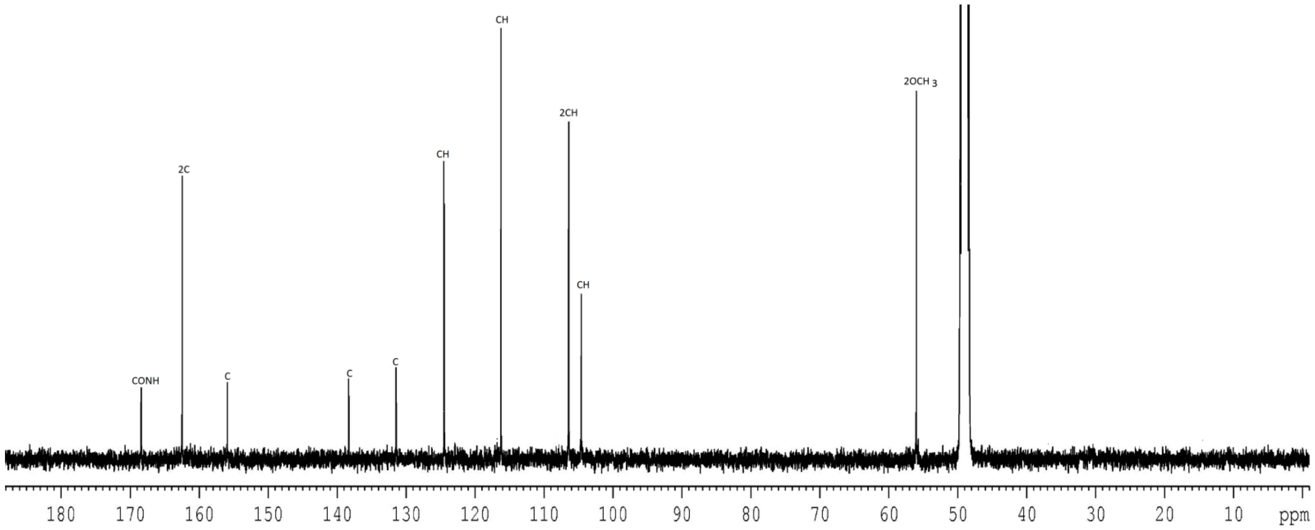

compound **8**

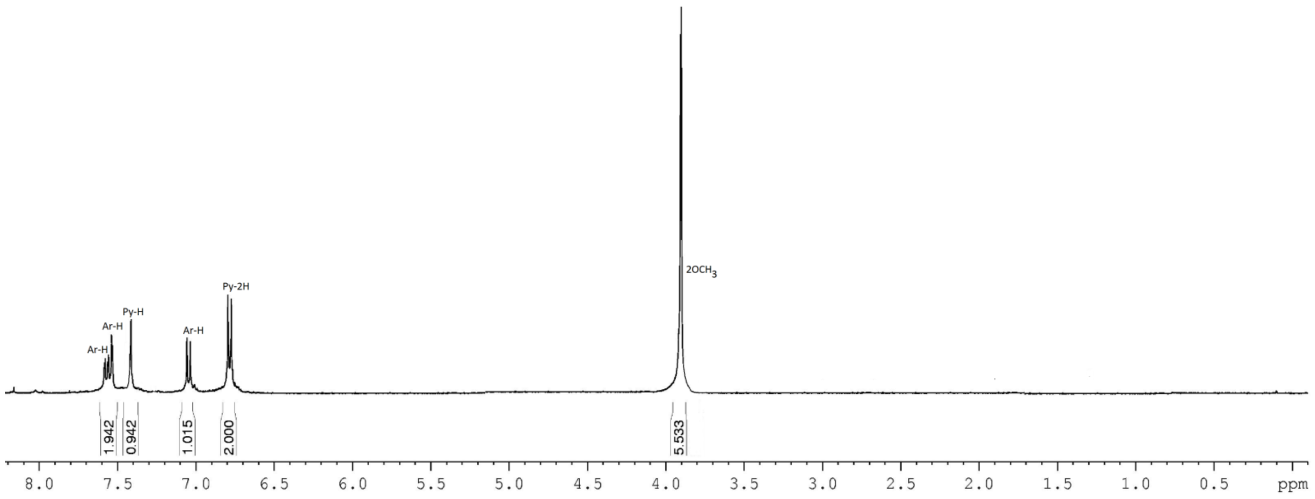

Ar-H
Ar-H
Py-H
Ar-H
Py-2H
2OCH3
1.942
0.942
1.015
2.000
5.533
8.0 7.5 7.0 6.5 6.0 5.5 5.0 4.5 4.0 3.5 3.0 2.5 2.0 1.5 1.0 0.5 ppm

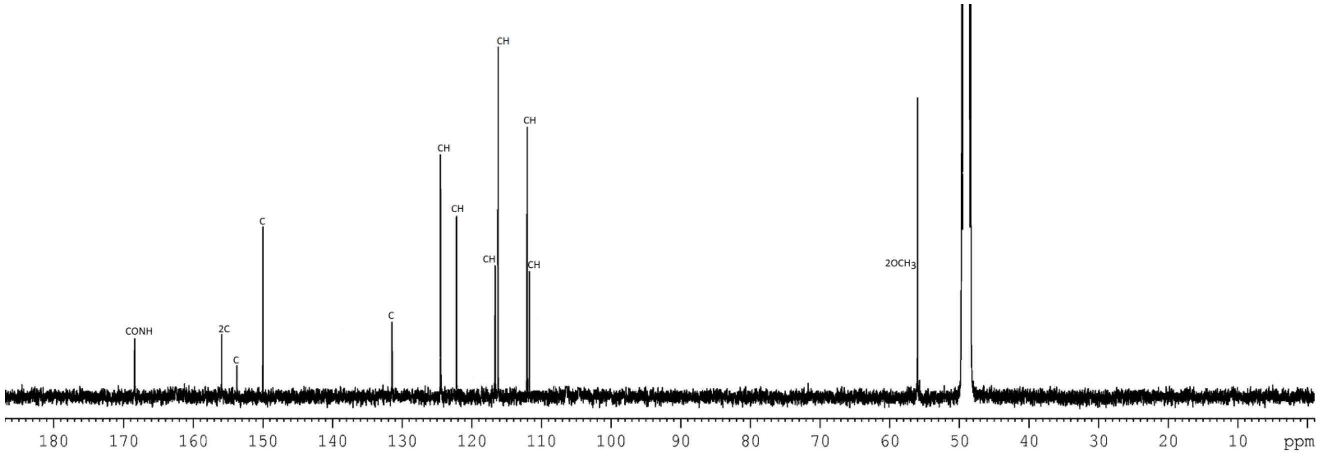

CONH
2C
C
C
C
CH
CH
CH
CH
CH
CH
2OCH3
180 170 160 150 140 130 120 110 100 90 80 70 60 50 40 30 20 10 ppm

compound **9**

1H

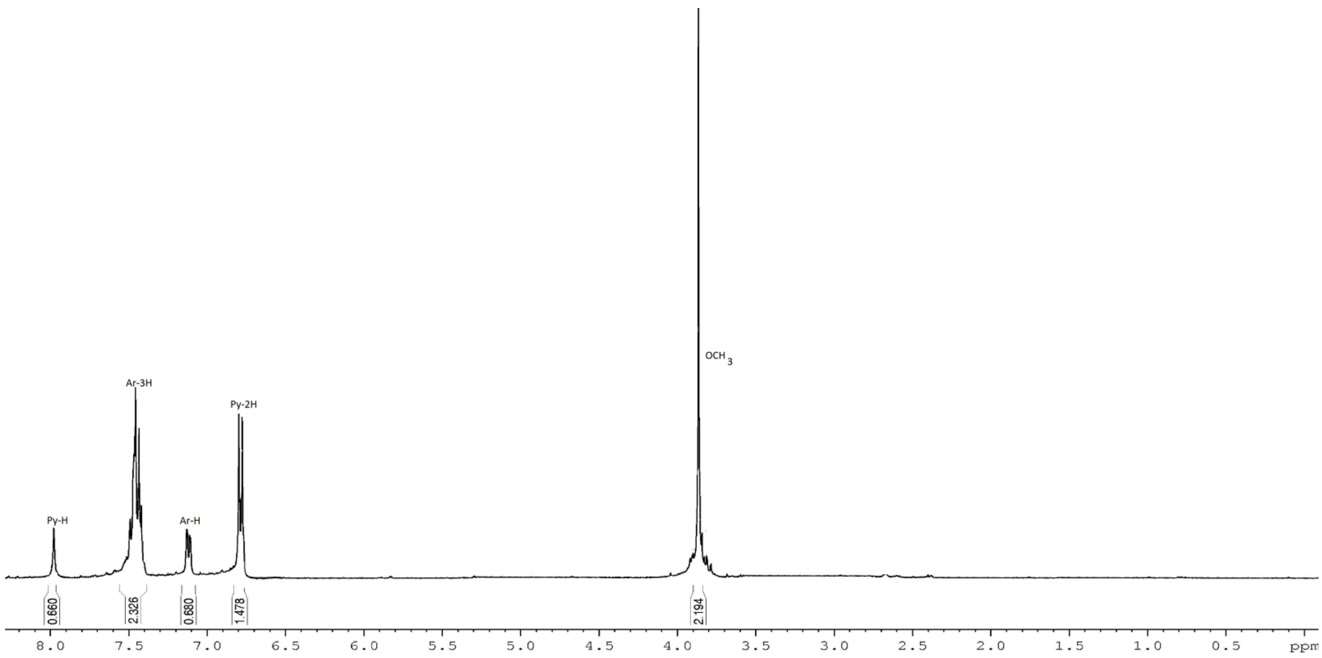

13C

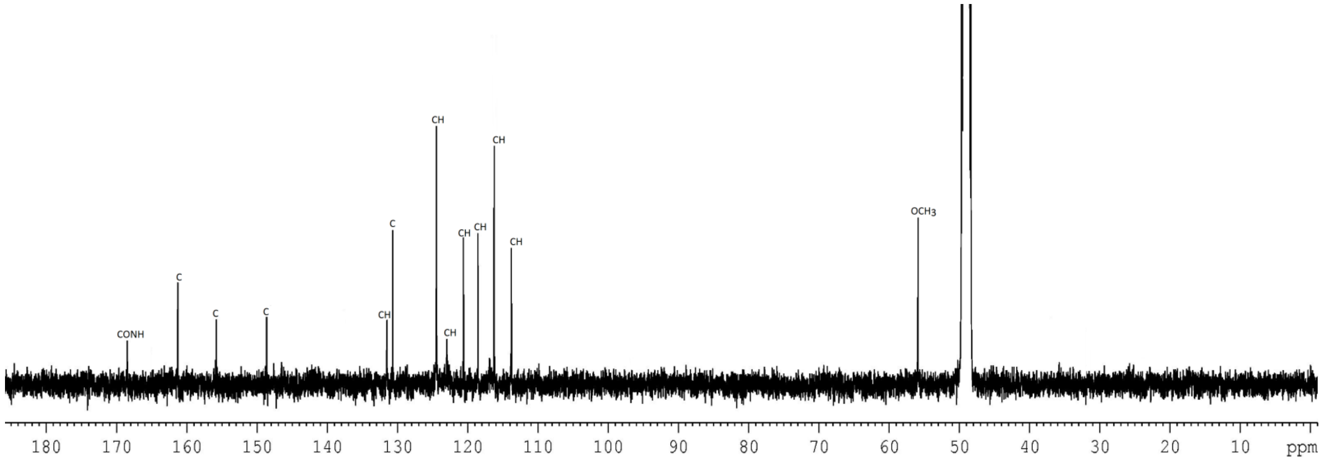

compound **10**

1H

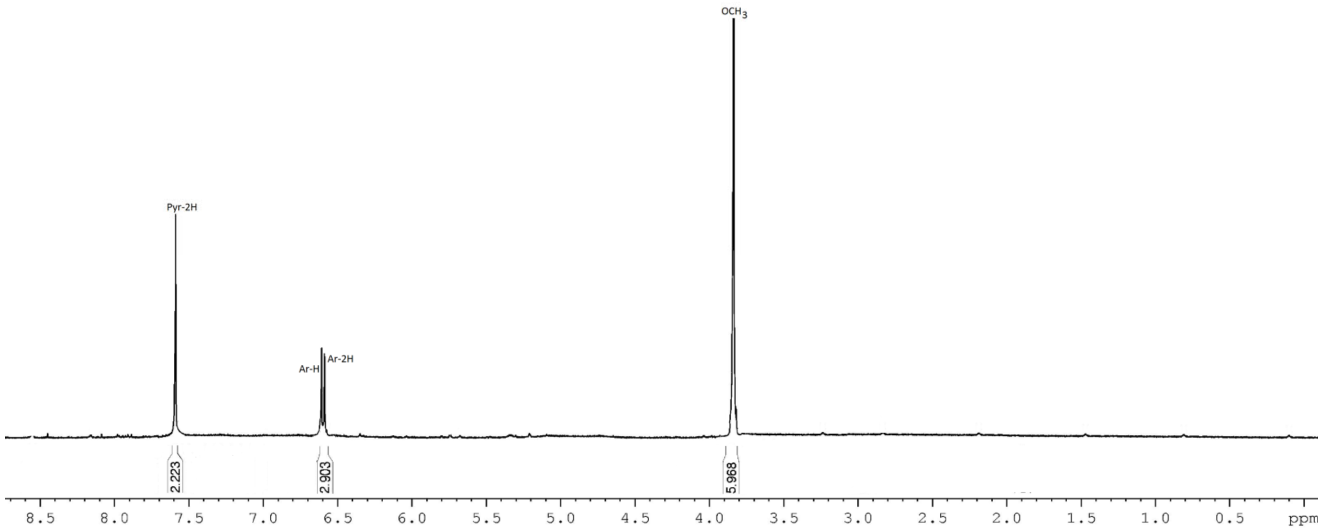

13C

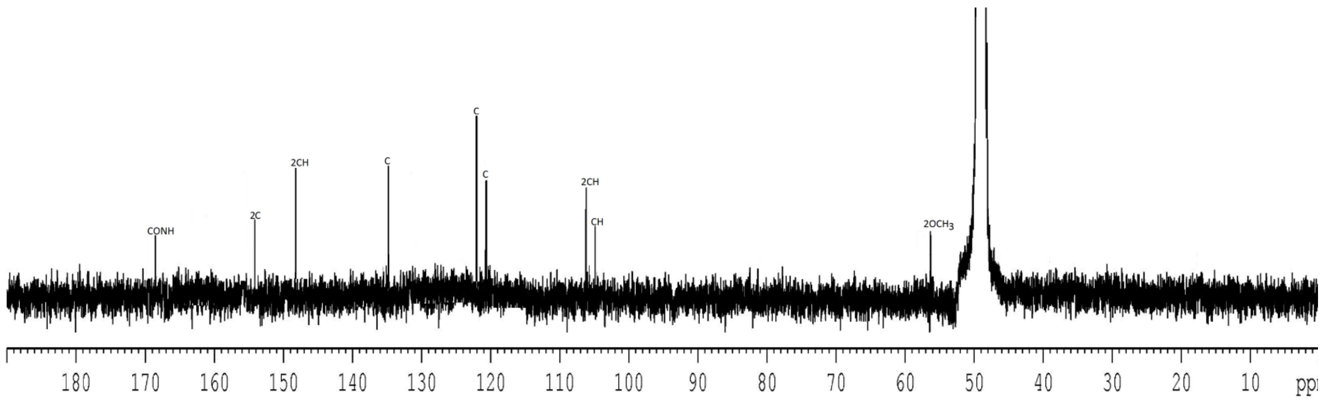

compound **11**

1H

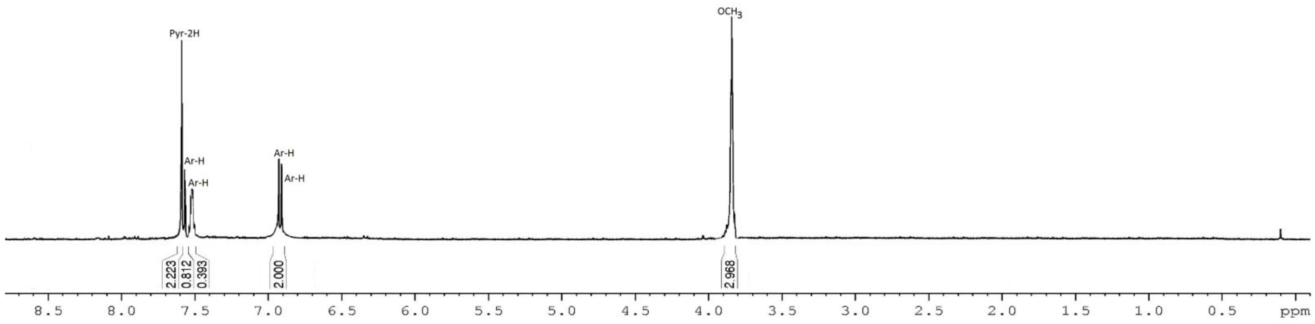

13C

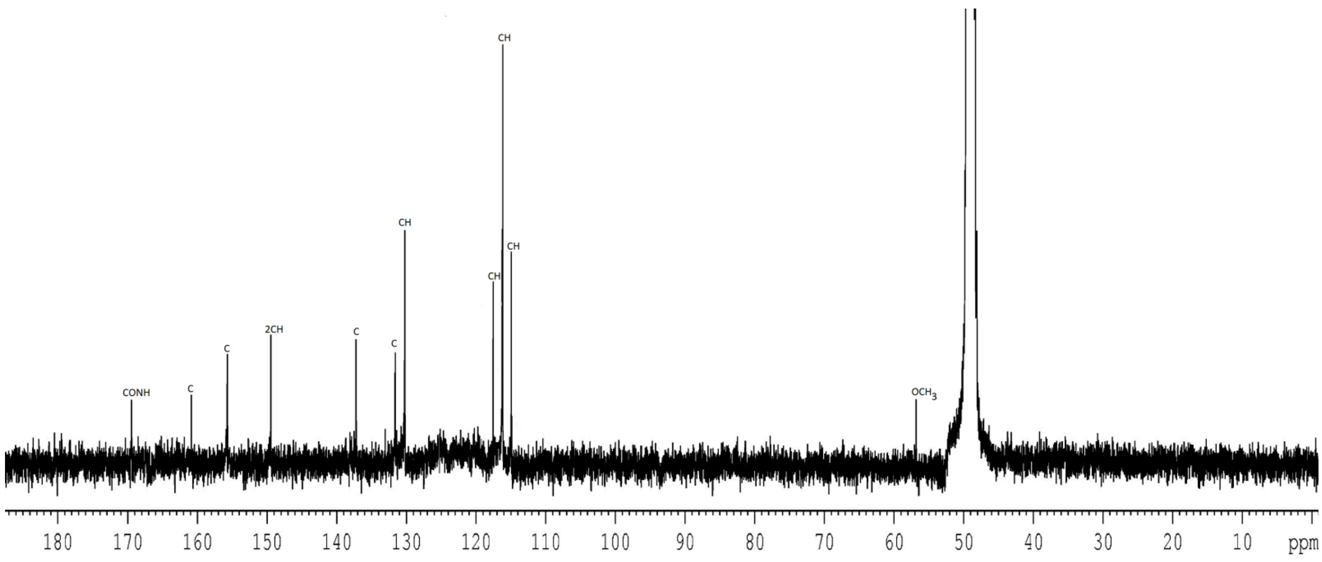

compound **12**

1H

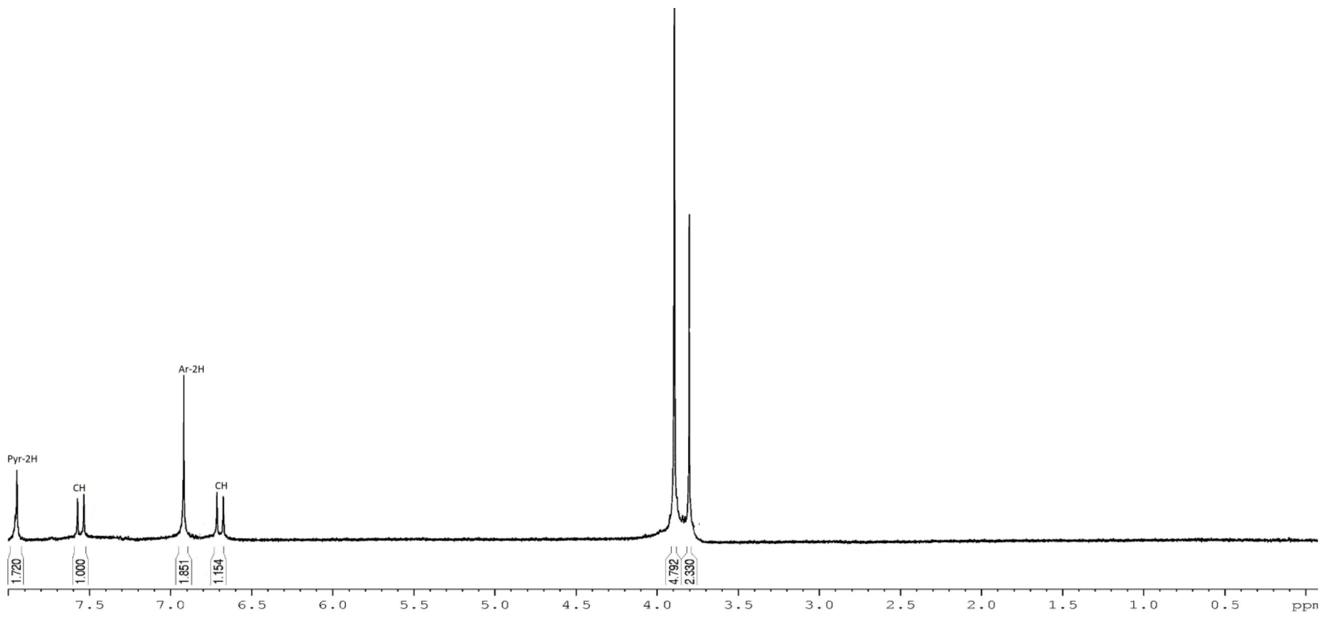

13C

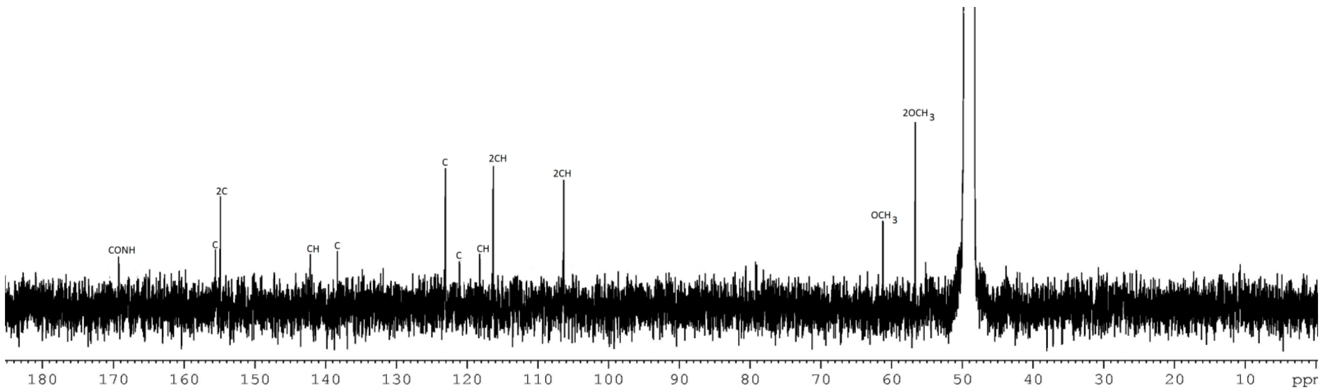

compound **13**

1H

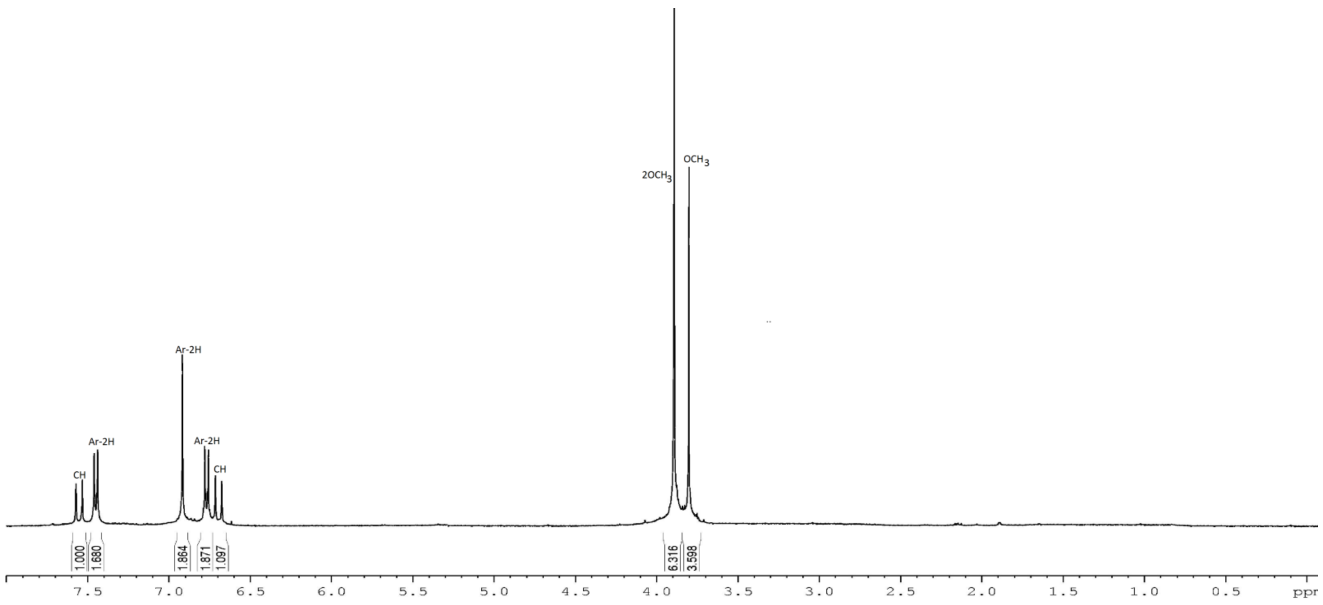

13C

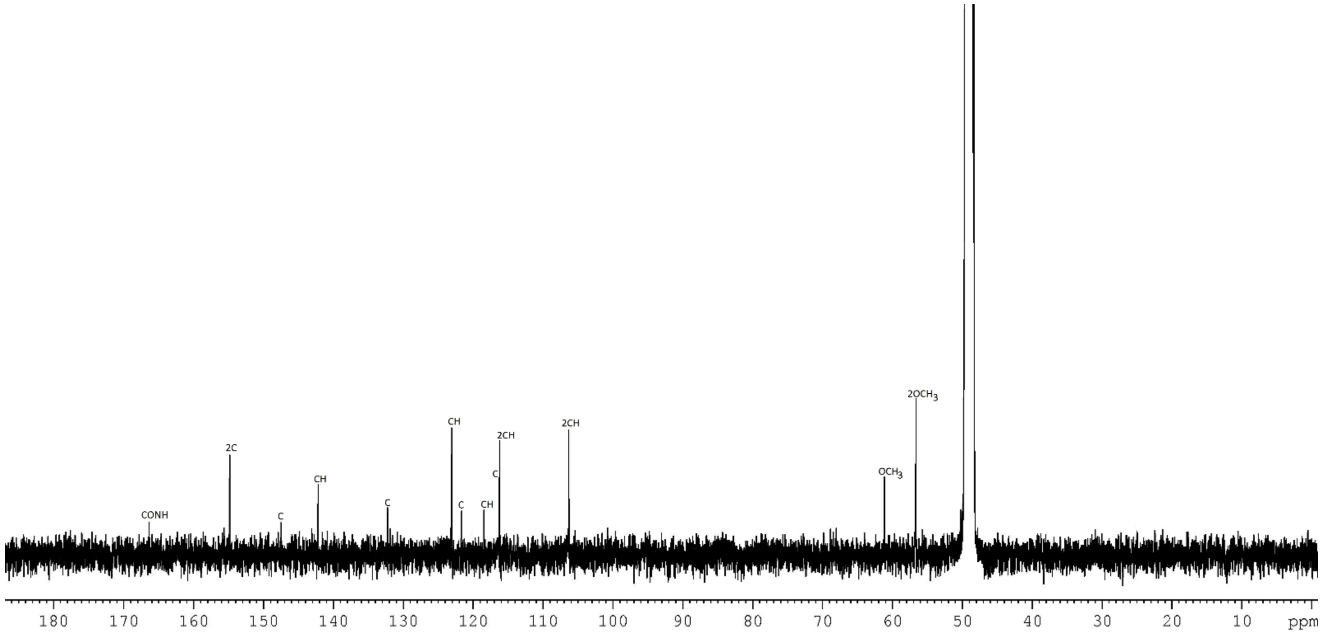

compound **14**

1H

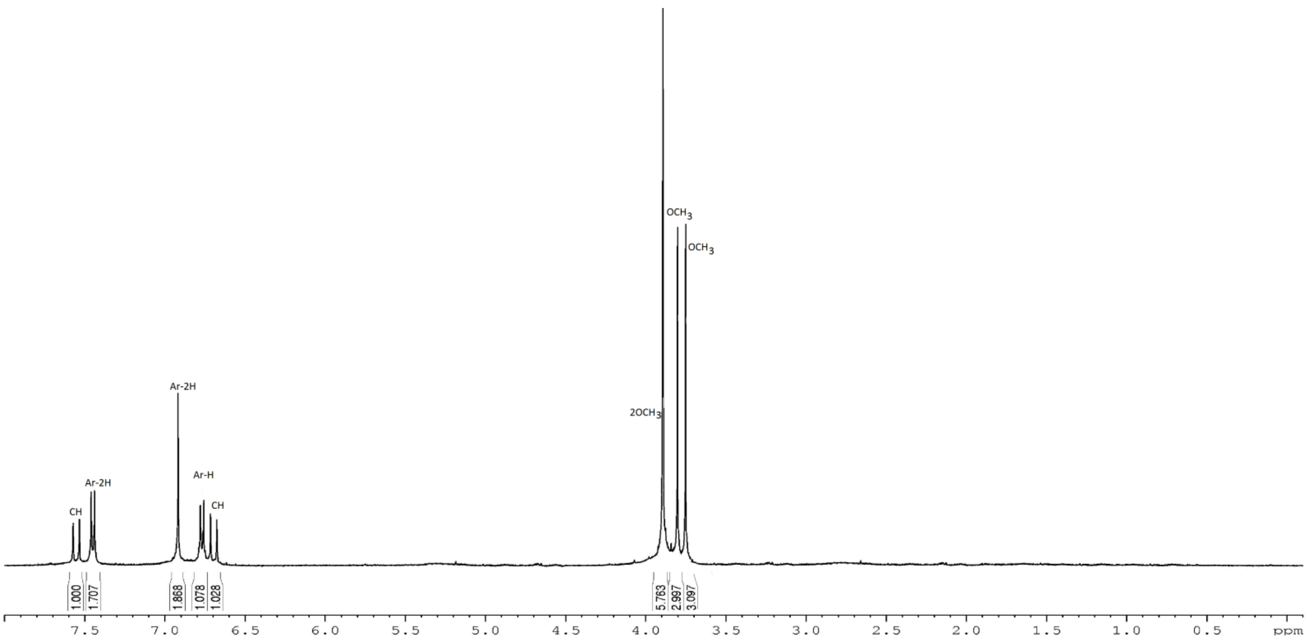

13C

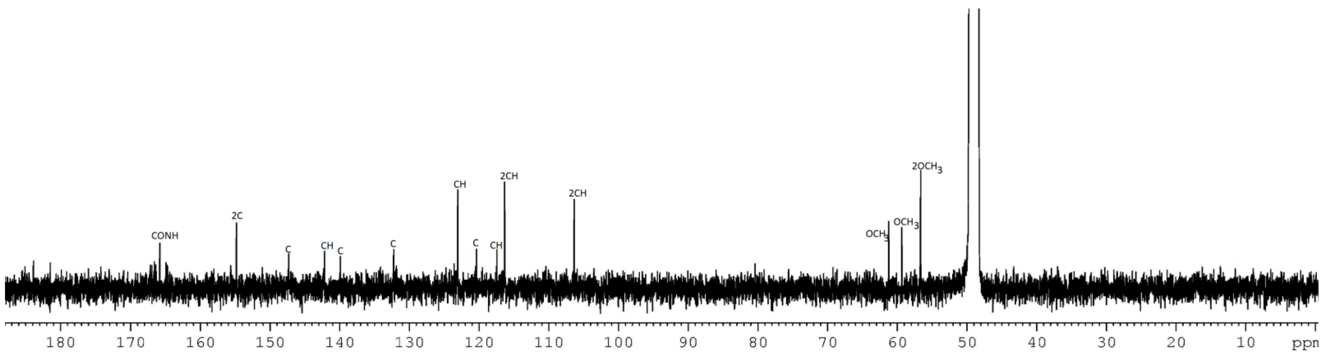

compound **15**

[1]H

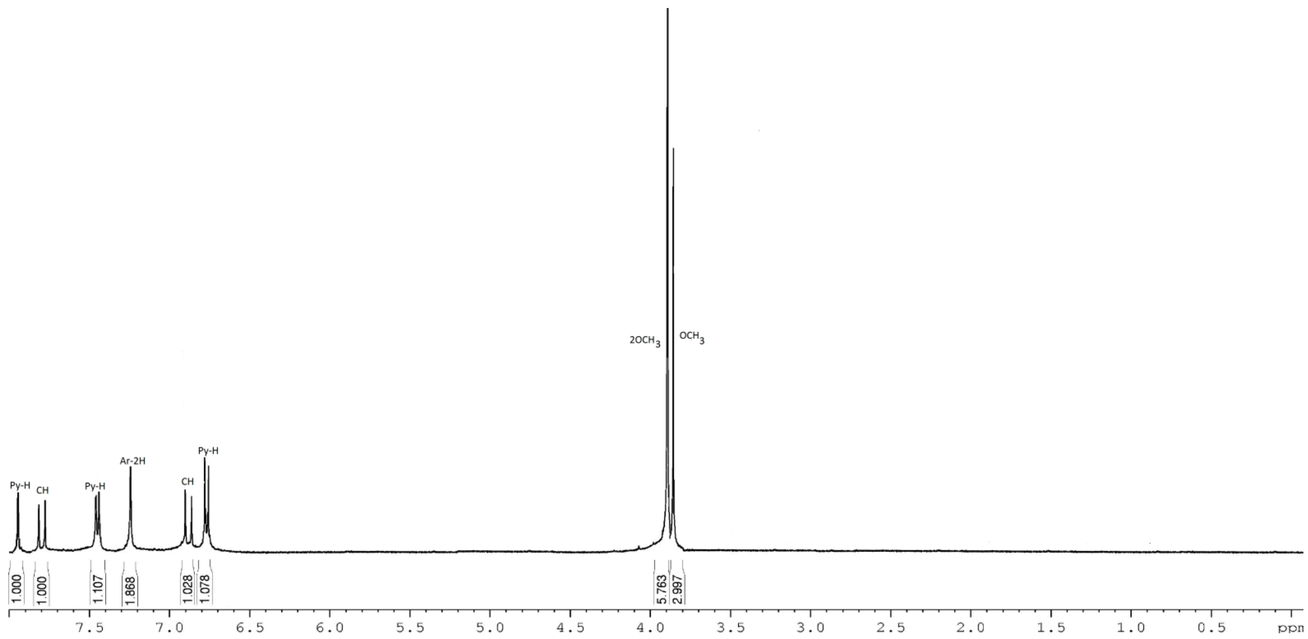

[13]C

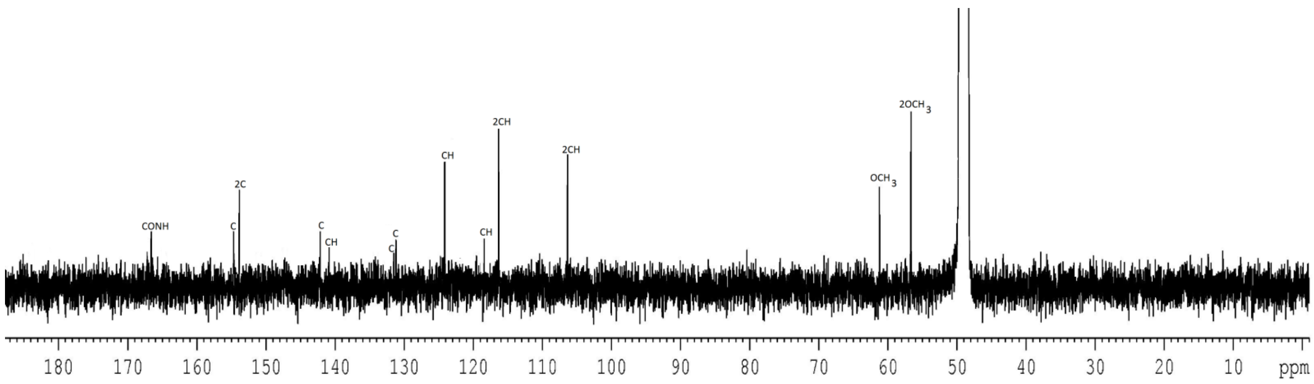

compound **16**

1H

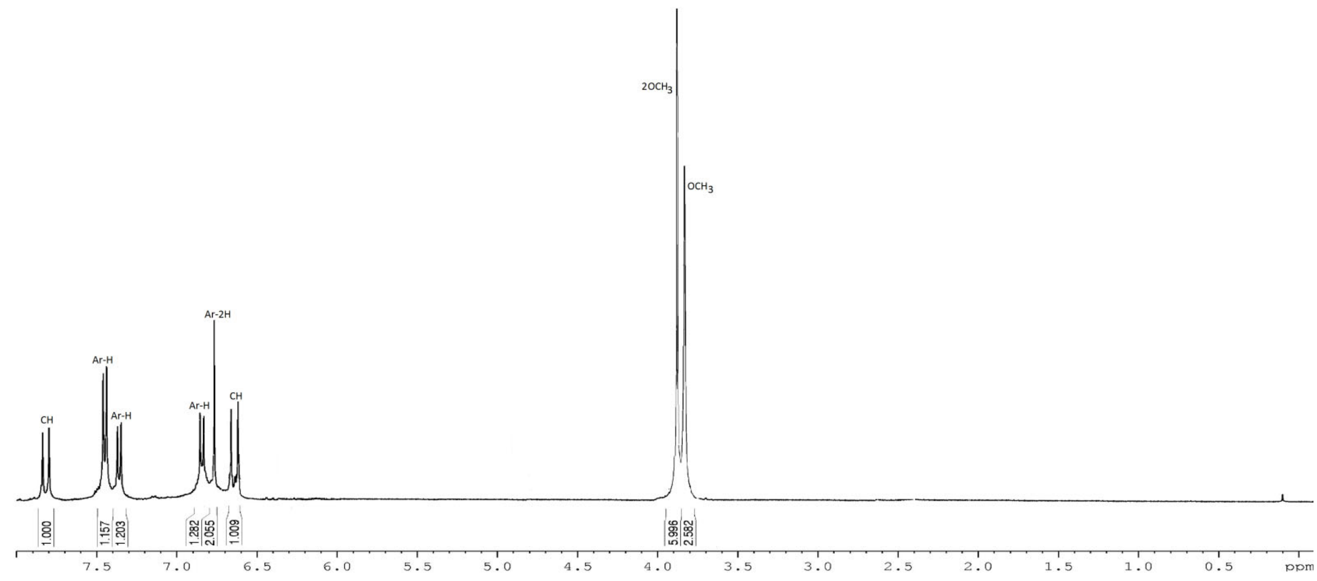

13C

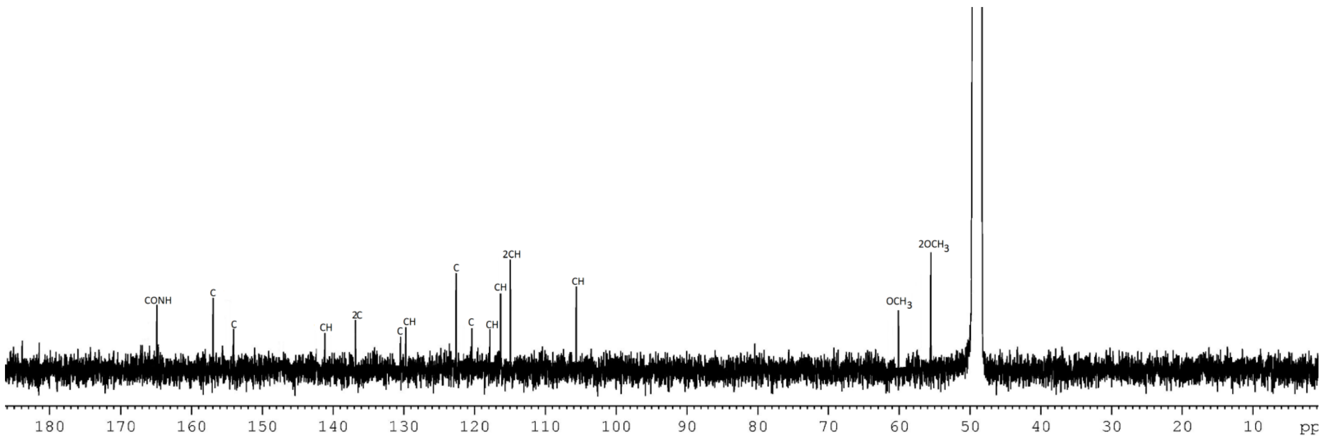

compound **17**

1H

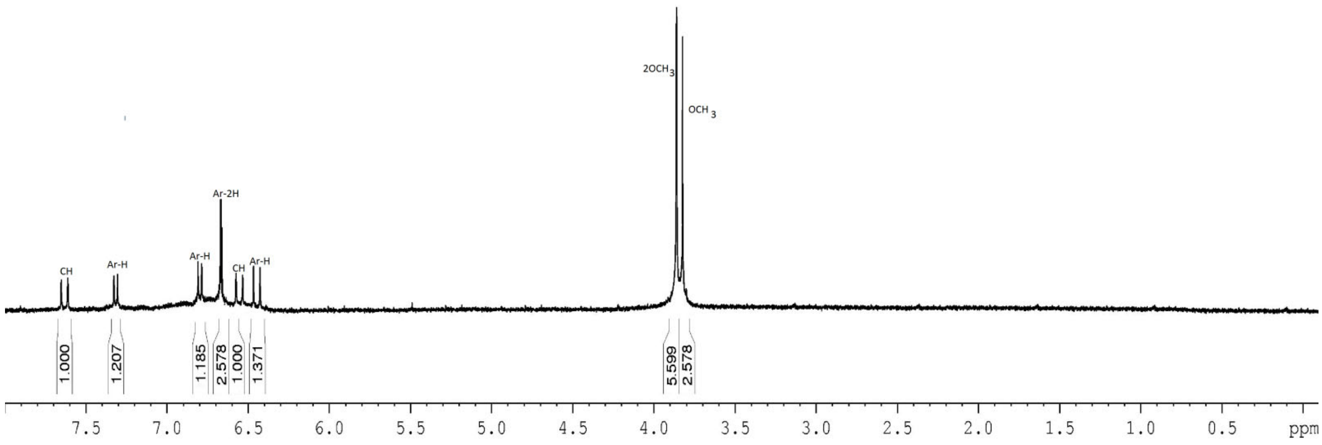

13C

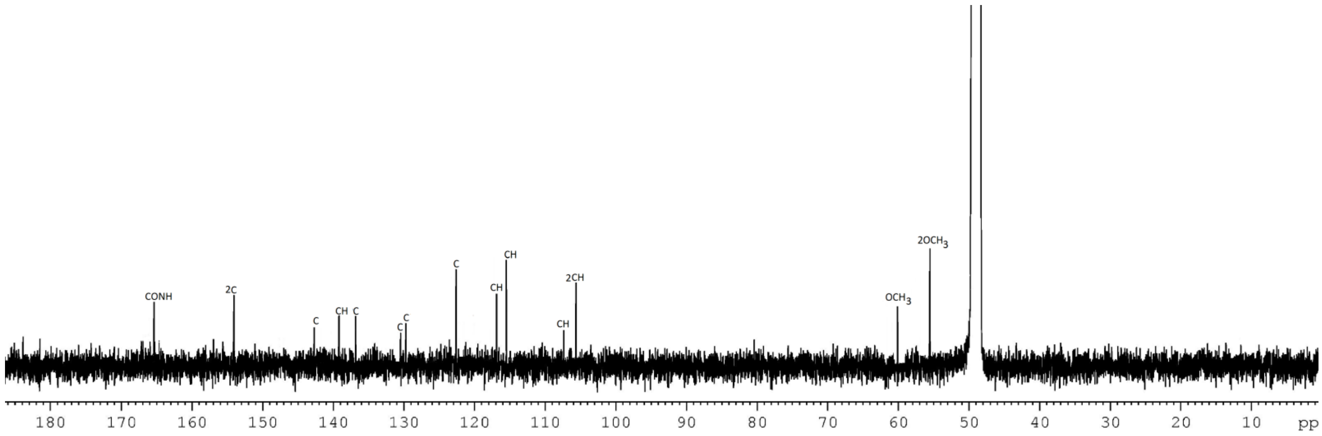

compound **18**

1H

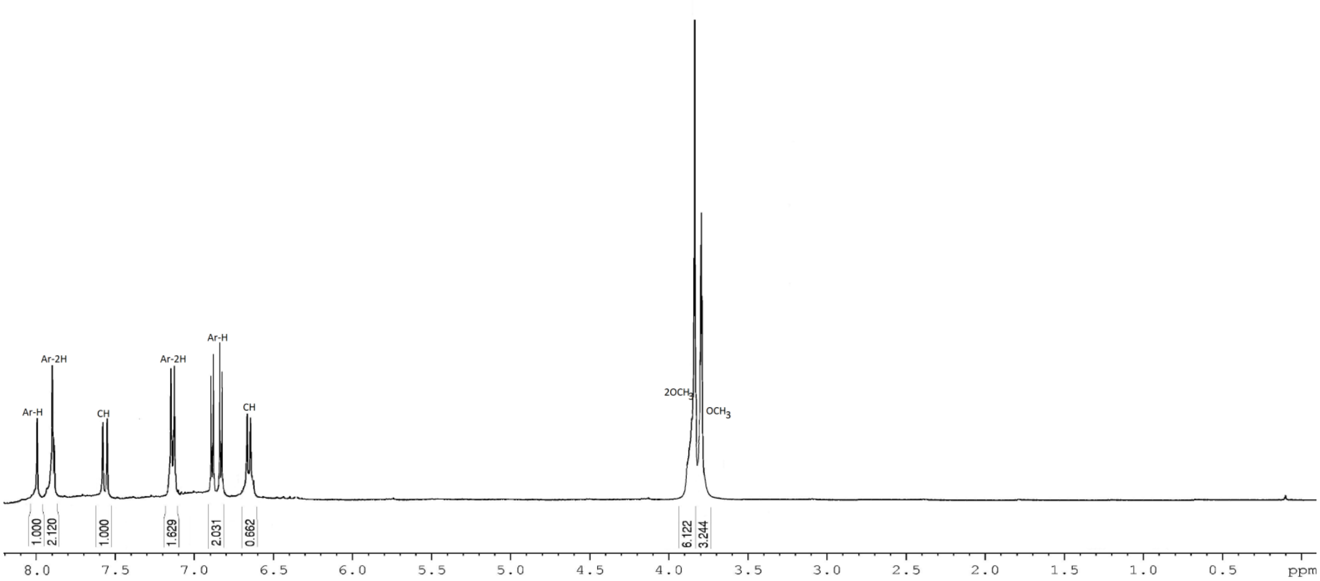

13 C

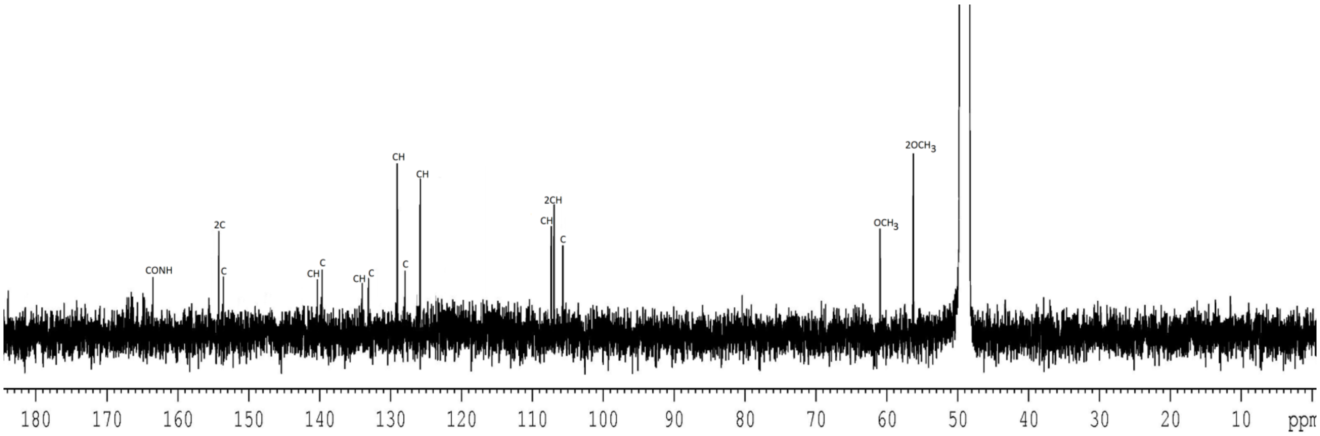

Supplement: Supplementary file 1 [file ijms-22-03685-s001.pdf]
